# Overview of mechanisms and novel therapies on rheumatoid arthritis from a cellular perspective

**DOI:** 10.3389/fimmu.2024.1461756

**Published:** 2024-09-23

**Authors:** Peng Han, Xiaoying Liu, Jiang He, Luyang Han, Jinyao Li

**Affiliations:** ^1^ Xinjiang Key Laboratory of Biological Resources and Genetic Engineering, College of Life Science and Technology, Xinjiang University, Urumqi, China; ^2^ Key Laboratory of Uygur Medicine, Xinjiang Institute of Materia Medica, Urumqi, China

**Keywords:** autoimmunity, rheumatoid arthritis, therapeutic strategies, inflammation, new treatment methods

## Abstract

Rheumatoid arthritis (RA) is an autoimmune disease characterized by synovial inflammation of joints in response to autoimmune disorders. Once triggered, many factors were involved in the development of RA, including both cellular factors like osteoclasts, synovial fibroblasts, T cells, B cells, and soluble factors like interleukin-1 (IL-1), IL-6, IL-17 and tumor necrosis factor-α (TNF-α), etc. The complex interplay of those factors results in such pathological abnormality as synovial hyperplasia, bone injury and multi-joint inflammation. To treat this chronic life-affecting disease, the primary drugs used in easing the patient’s symptoms are disease-modifying antirheumatic drugs (DMARDs). However, these traditional drugs could cause serious side effects, such as high blood pressure and stomach ulcers. Interestingly, recent discoveries on the pathogenesis of RA have led to various new kinds of drugs or therapeutic strategies. Therefore, we present a timely review of the latest development in this field, focusing on the cellular aspects of RA pathogenesis and new therapeutic methods in clinical application. Hopefully it can provide translational guide to the pre-clinical research and treatment for the autoimmune joint disease.

## 1 Introduction

**Figure 1:**
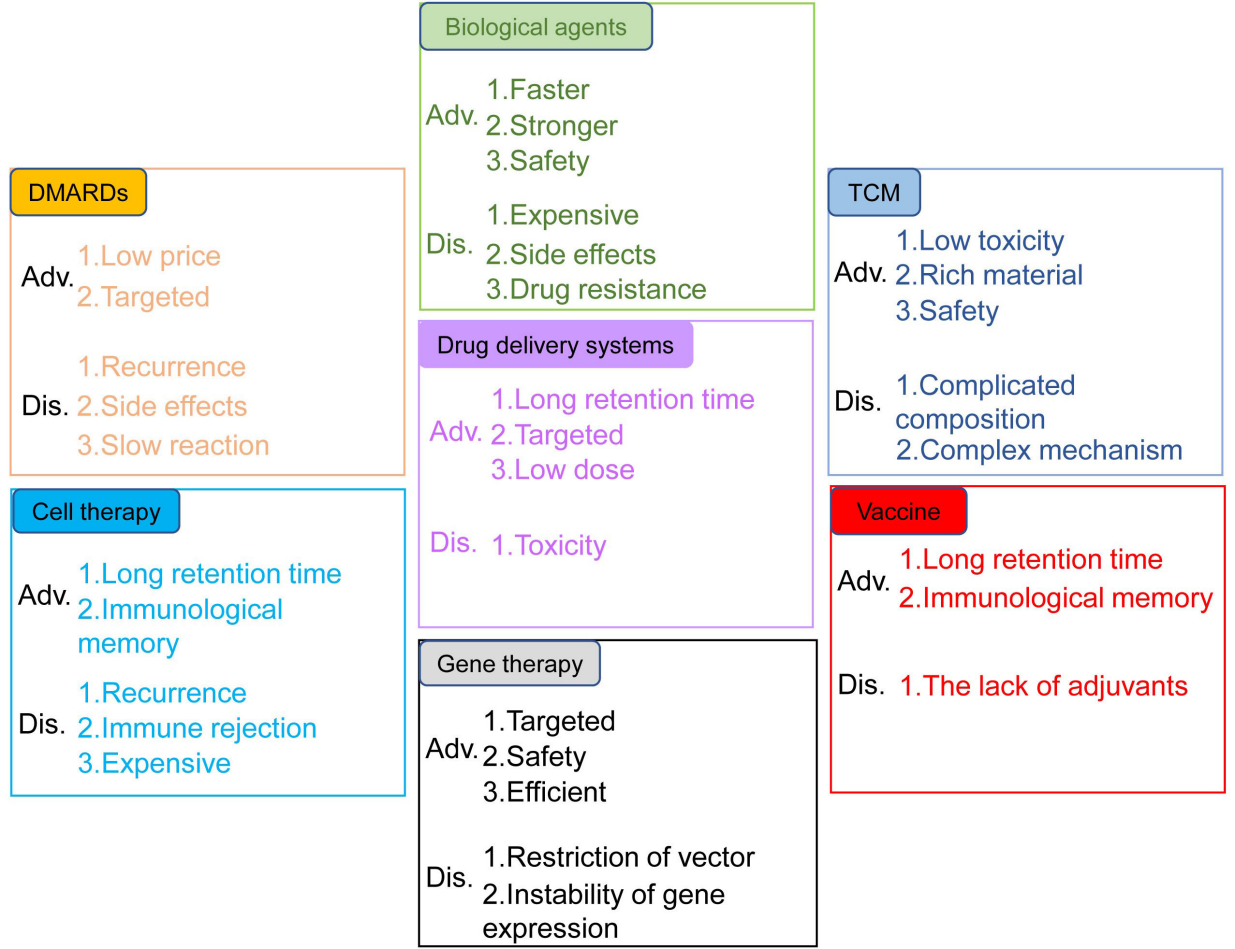
Advantages and disadvantages of the treatments for RA. The treatments include DMARDs, biological agents, TCM, drug delivery systems, cell therapy, gene therapy and vaccines.

Rheumatoid arthritis (RA) is a chronic, inflammatory, persistent and systemic autoimmune disease. According to the statistics from World Health Organization, RA is one of the most challenging diseases for both the physicians and patients (1). RA, featured by the destruction of bone, synovial inflammation and pannus formation, results in irreversible tissue injury targeting tendons, cartilage and bone (2). It has been confirmed that the genetic susceptibility of major histocompatibility complex class II (MHC II) alleles has strong correlation with RA, and twin studies also highlighted the important role of environmental factors in the pathological progression of RA (3). Auto-antibodies, inflammatory cytokines and various immune cells including T cells, dendritic cells (DCs) and macrophages were also involved in RA pathogenesis (4). Conventional synthetic disease-modifying antirheumatic drugs (DMARDs) have been used in the clinical treatment of RA, such as methotrexate, cyclophosphamide and non-steroidal anti-inflammatory drugs (NSAIDs) (5). However, these drugs cause such serious side effects, such as high blood pressure and stomach ulcers, etc. New therapies for RA, including biological agents, traditional Chinese medicine, cell transfer, gene therapy, vaccines, and new drug delivery systems, have their own advantages and disadvantages (**Figure 1**).

## 2 Pathogenesis of RA

RA is an inflammatory disease caused by multi-cells communication and interaction by cytokines, which result in a variety of pathological reactions (6). The induction and progress of RA require the participation of osteoclasts, synovial fibroblasts, T cells, B cells, natural killer (NK) cells and other cells. These immune cells can be activated through nuclear factor kappa-B (NF-κB), mitogen-activated protein kinase (MAPK), phosphoinositide 3-kinase-protein kinase B (PI3K-Akt) and Janus kinase- signal transducer and activator of transcription (JAK-STATs) signaling pathways to secrete inflammatory cytokines such as IL-1, IL-6, IL-17, tumor necrosis factor (TNF)-α and so on (7).

**Figure 2:**
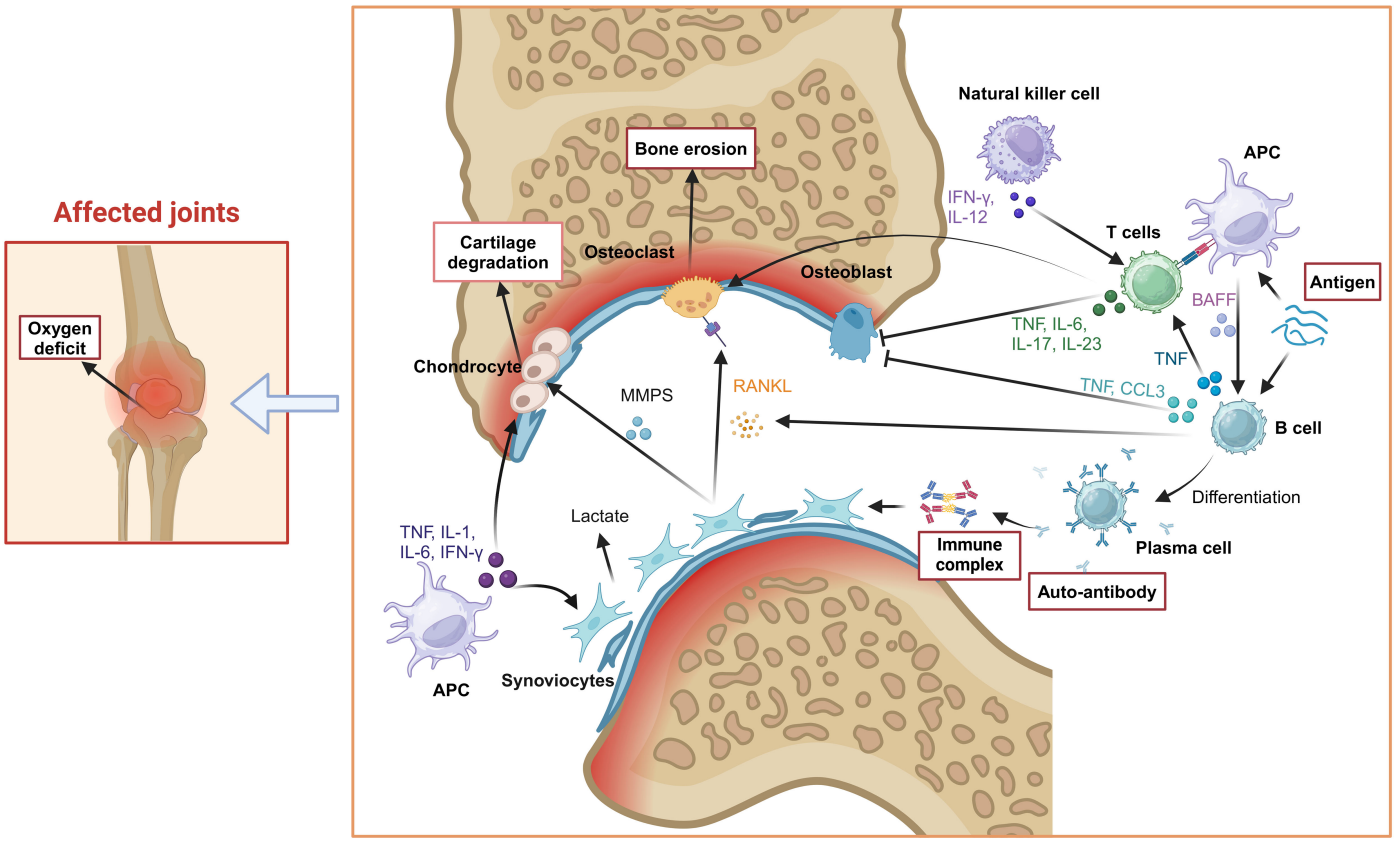
Pathogenesis of RA. Tissue cells (osteoclasts, synovial fibroblasts), immune cells (T cells, B cells, NK cells), inflammatory cytokines (IL-1, IL-6, IL-17, TNF-α) and other factors (RANKL) were involved in the occurrence and progress of RA, which induced joint inflammation and causes bone and joint injury. Created with BioRender.com.

The cell-cell contact or cytokine signals from antigen-presenting cells (APCs) (especially Dendritic cells, DC) induced the conversion of T cells, B cells, synovial fibroblasts, osteoclasts and osteoblasts. At the early stage of RA, DCs mature through a series of complex physiological processes, such as specific signal transduction and transcriptional programming. Under the stimulation of cytokines secreted by mature DCs (mDCs), T cells and B cells polarized into inflammatory subsets. For example, IL-12 induces the differentiation of CD4^+^ T cells into Th1 cells, whereas upon co-stimulation with IL-6 and TGF-β, CD4^+^ T cells differentiate into Th17 cells. Likewise, B-cell activating factor (BAFF) secreted by mDCs induces the activation and differentiation of B cells. Furthermore, these inflammatory cells migrate into the joint tissues, secrete inflammatory cytokines, aggravate joint inflammation, and therefore cause tissue lesions. Th17 cells induce the formation of osteoclasts by producing IL-17 and promote bone resorption by up-regulating the receptor activator of Nuclear Factor-κB Ligand (RANKL) (8). B cells inhibit osteoblasts differentiation by producing TNF-α and CCL3 in RA patients (9). On the other hand, when exposed to TNF-α, IFN-γ, and IL-1β secreted by mDCs in synovial fluid, synovial fibroblasts release multiple matrix metalloproteinases (MMPs) that degrade articular cartilage and bone tissue (10). Moreover, the RANKL released by synovial fibroblasts breaks the balance of osteoclasts and osteoblasts, and a large number of osteoclasts develop and mature, which further lead to calcification of bone and joint. Therefore, effective inhibition of these cell-induced inflammation spread in the earliest possible time could block the structural damage of the joints in the long run for the treatment of RA (**Figure 2**).

### 2.1 Synovial fibroblasts

Synovial fibroblasts play an active role in the pathogenesis of RA by undergoing various biological processes such as signal transduction, gene regulation and metabolism. The healthy synovial tissue is usually free of inflammatory cells, whereas in the diseased state, however, the influx of inflammatory cells and their release of inflammatory factors lead to the diseased of synovial fibroblasts. Stimulated by TNF-α and IL-1, RANKL secreted by synovial fibroblasts induces macrophages to transform into osteoclasts (11). In addition, IL-17A and TNF-α activate ELF3, inducing synovial fibroblasts to synthesize and secrete MMPs (12). In addition to these signals, macrophage derived microvesicles (METs) from THP-1 cells activate the PI3K/Akt signaling pathway through the receptor of the DNA sensor GMP-AMP synthase (cGAS), significantly upregulating TNF, IL-1β, and MMPs (13). Activation of NF-κB induces MMPs expression in synovial fibroblasts (14, 15). MMP-1 preferentially degrades fibrous collagen, while MMP-3 degrades a wide range of extracellular matrixes (16–18). In the inflammatory joint, the vascular endothelial growth factor (VEGF) directly targets the vascular endothelium of synovial tissue, stimulates endothelial cell proliferation, increases microvascular permeability, and leads to vascular membrane formation and tissue injury (19, 20). Furthermore, transforming growth factor β (TGF-β) in joints induces IL-6 and VEGF production in synovial fibroblasts by activating NF-κB (21), which forms a feedback in the synovial tissue to continuously promote the formation of pannus.

Another major reason for fibroblast-induced disease in synovial tissue is that these cells are resistant to apoptosis (23), which is related to their high expression of anti-apoptotic molecules such as Fas-associating protein with a novel death domain- like ICE inhibitor protein (FLIP) and C-C motif chemokine ligand 5 (CCL5). FLIP exerts its anti-apoptotic effect through inhibition of Fas-mediated apoptosis (24). The strong expression of CCL5 and C-X-C motif chemokine ligand 9 (CXCL9/10) has been observed at mRNA and protein levels in synovial cells of RA (25). Moreover, silencing C-C chemokine receptor type 5 (CCR5) can promote synovial cell apoptosis and decrease the release of inflammatory factors in CIA rats by inhibiting MAPK-related signaling pathways (26). Apoptosis resistance of fibroblast-like synovial cells in RA, in turn, promotes pannus formation, induces cartilage and bone destruction and protects infiltrating cells from apoptosis (27).

Synovial hyperplasia induced by metabolic conversion may originate from signaling. For example, IFN-α activates the TLR3 and TLR7 signaling pathway in RASFs (28). IRAK4 is a pivotal enzyme in the Toll-like receptor (TLR)/MyD88 dependent signaling pathway. Inhibition of IRAK4 activity can rebalance metabolic disorders by reversing the metabolism of RASFs (29). Similarly, as an effector downstream of TLR7, HIF enhances glycolysis by upregulating the expression of glucose transporters (GLUT1 and GLUT3), transactivating genes responsible for O_2_ demand and mitochondrial activity in RASFs (29–31). Therefore, the hypoxic microenvironment in the joint cavity of RA patients induces mitochondrial dysfunction, resulting in reduced ATP production (32). RASFs as “tumor cell” in the destructive joint, proliferate rapidly through the Warburg effect. Besides, RASFs may undergo metabolic reprogramming to turn into “metabolic factories”, with its metabolite transforming into lactate (86.12%) and alanine (8.33%) from CO_2_ (99.96%) (30). Lactate affects the differentiation and activation of T cells, B cells and osteoclasts. Hence, the crosstalk between metabolite and synovial fibroblasts may act as a second signal of metabolic conversion in synovial fibroblasts, then aggravate the symptom of RA.

### 2.2 Osteoclasts and osteoblasts

**Figure 3:**
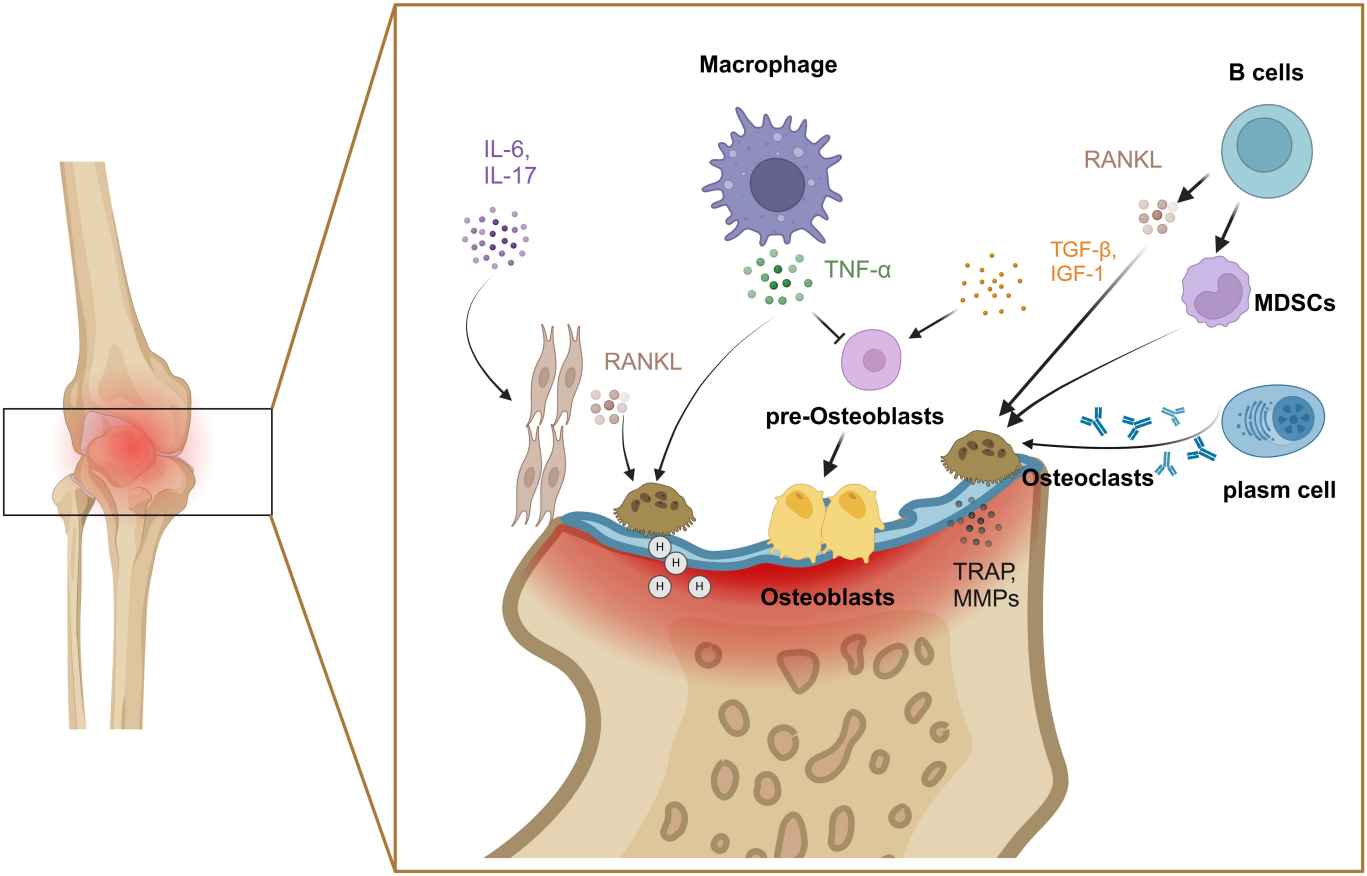
Mechanisms of bone injury by osteoclasts and osteoblasts. Various factors including IL-6, IL-17, TNF-α and RANKL were involved in the imbalance between osteoclasts and osteoblasts. Created with BioRender.com.

In RA, another cellular contribution comes from the imbalance between osteoclasts and osteoblasts as the primary factor of bone destruction. Bone remodeling relies on the balance between bone formation and destruction, which maintains bone homeostasis (33). Osteoclasts affect the process of bone resorption. The RANKL/RANK signaling pathway plays a crucial role in regulating the formation and differentiation of osteoclasts (34), which mediates the transcriptional activation of nuclear factor of activated T cells 1(NFATc1). The expression of NFATc1 is associated with osteoclast differentiation genes. In arthritic joints, the synovium is a common site for forming bone resorptive osteoclasts, stimulated by RANKL from synovium fibroblasts and B cells (35, 36). IL-6 and IL-17 induce RANKL expression to stimulate osteoclast differentiation by activating NF-κB and MAPK from their monocyte precursors (37, 38). TNF-α can increase the number of osteoclast precursor cells and induce osteoclast differentiation and maturation in bone marrow (39). MDSCs have recently been identified as precursor cells of osteoclasts, and are triggered by B cells, especially switched memory B cells (40). Anti-cyclocitrullinate peptide antibody (ACPA) from plasma cells contributes to osteoclast differentiation and activation by stimulating osteoclast precursors via the Fcγ receptor (41). Mature osteoclasts bind to bone tissue by recognizing hydroxyapatite (42), vitronectin and its receptor αvβ3 (43). Furthermore, osteoclasts lower the pH value of the binding zone, and secret a large number of matrix degrading enzymes, such as MMPs and tartrate-resistant acid phosphatases, to facilitate the degradation of collagen and bone (44). Meanwhile, osteoclasts modulate their functional activity through the endocytosis of degradation products, thereby promoting the continuous degradation of bone tissue and facilitating the mineralization and deposition of bone tissue (**Figure 3**).

Bone marrow mesenchymal stem cells differentiate into osteoblast progenitors under the stimulation of cytokines such as insulin-like growth factor 1 (IGF-1) and TGF-β (45). Studies have found that the activation of Wnt/β-catenin signaling pathway in osteoblasts of RA patients was increased, which inhibited osteoblast activity and promoted osteoblast apoptosis, primarily mediated by TNF-α and IL-1 (46). In addition, the joint microenvironment also inhibits osteoblast activity and erosion repair once RA has developed (47–49). As early as 1986, pioneering researchers reported that monocyte-derived TNF-α directly inhibited the synthesis of collagen in osteoblasts (50). Furthermore, IL-17 from Th17 cells promotes collagen degradation and inhibits both collagen synthesis and bone formation by interfering with osteoblast function (**Figure 3**) (51).

### 2.3 T cells

**Figure 4:**
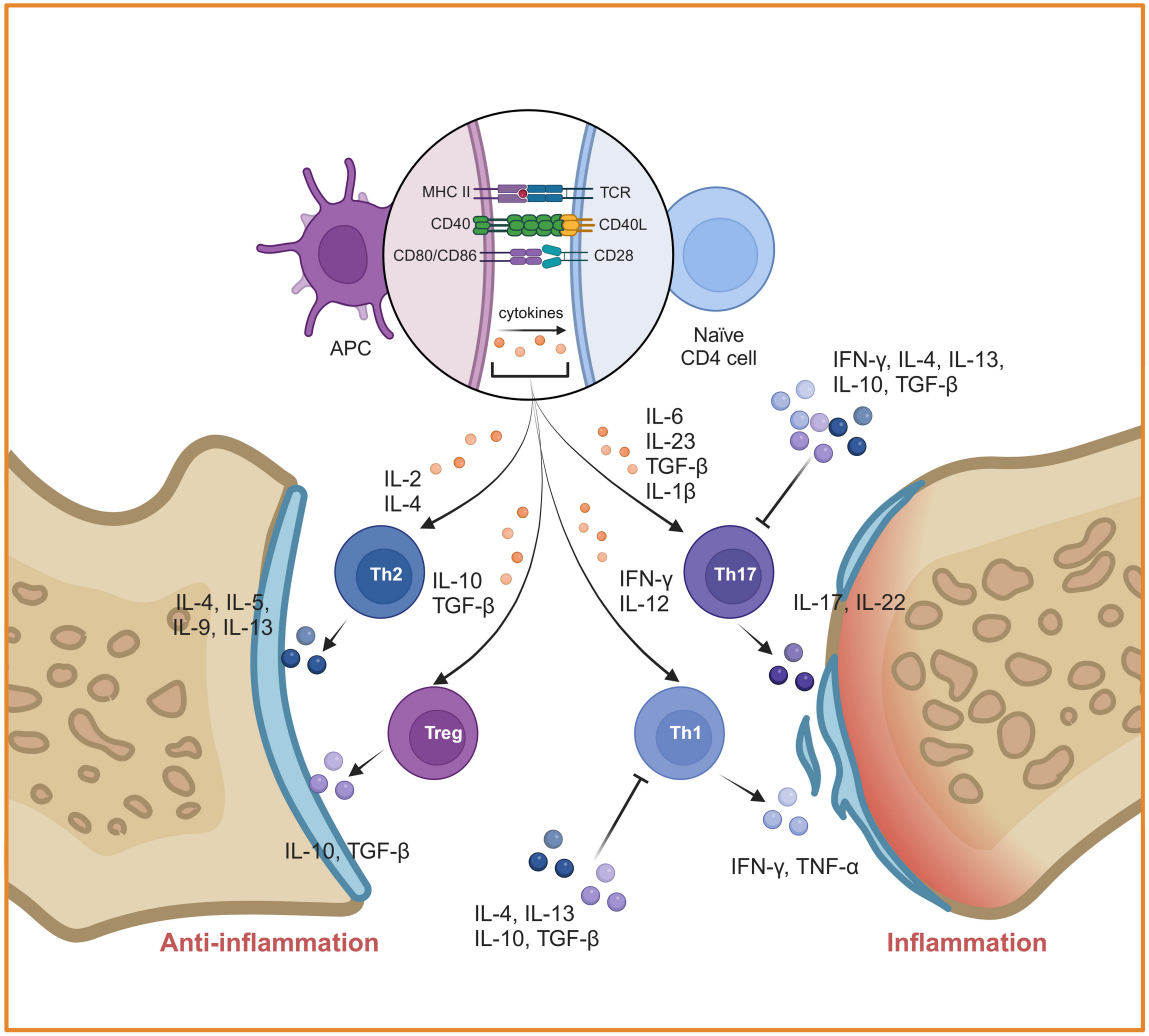
Mechanisms of inflammation mediated by CD4^+^ T cells in RA. CD4^+^ T cells were activated by DCs and differentiated into different subtypes. The imbalances of Th1/Th2 and Th17/Treg play a key role in RA. Created with BioRender.com.

Autoreactive T cells that have escaped negative selection in thymus are the third cellular source for the joint inflammation and destruction (52). Different subtypes of CD4^+^ T cells play an important role in the induction and progress of RA, especially the imbalance of Th1/Th2 and Th17/Treg (**Figure 4**). Th1 and Th17 cells secrete a great deal of inflammatory cytokines such as Interferon-gamma (IFN-γ), IL-17A and so on, which could lead to monocyte activation, osteoclast formation, synovial cell proliferation and vascular formation. It is proven that IFN-γ secreted by Th1 can block Th17 development (53). Under autoinflammatory conditions, T cell plasticity can lead to a shift between different Th cell subsets. For example, Th17 cells are identified as a “plastic” subset that can differentiate into other effector types (54). Th17 cell-derived “Th1-like” cells exhibit greater pathogenicity characterized by increased production of proinflammatory cytokines and enhanced proliferative capacity compared to classical Th17 or Th1 cells (55). The abnormal activated Th17 cells are produced rapidly and play a role in the early stage of inflammation, whereas Th1 cells exert a leading role in prolonging or promoting tissue inflammation during the later stage (56–58). Therefore, the “Th1-like” cells may serve a bridging function in the pathogenesis of RA. In contrast, Th2 cells and Tregs exert immunosuppressive effects during the disease process. Some studies have reported that Th2 cell-related cytokines including IL-4 and IL-13 are highly expressed in the synovial fluid of early RA patients, functioning as anti-inflammatory cytokines that inhibit the differentiation of Th1 and Th17. The Th17/Th2 subset has been identified in mice, which act as a pro-inflammatory phenotype through the secretion of IL-17A (59). However, the proportion of Th2 cells in joint are lower than blood from patients with active RA (60). This suggests that Th1 cells inhibit the differentiation and function of Th2 cells in the later stage of RA. Similarly, CD4^+^ Tregs inhibit the activation and proliferation of autoreactive T cells through direct contact or the secretion of inhibitory cytokines such as IL-10 and TGF-β. However, it has recently been found that Th1-like Treg cells, the dominant Treg cell subpopulation in RA, display low expression of TGF-β and defective inhibitory function (61). Notably, under the stimulation of IL-1β, IL-2, IL-21, IL-23 and human serum, Tregs can be converted into Th17-like Tregs, while controlling autoimmunity and inflammation (62).

Of note, cytotoxic CD8^+^ T cells are present in RA synovium, where they target citrullinated antigens and mediate joint tissue destruction (63). Another phenotype of CD8^+^ T is IL-17A^+^CD8^+^ T(Tc17) cells, similar with Th17 and CD8^+^ cells. Therefore, Tc17 can induce the expression of IL-6 and IL-8 by synovial fibroblasts and can be inhibited by anti-TNF-α and anti-IL-17A agent (64). Moreover, Li et al (65) have recently identified a high frequency of KIR^+^CD8^+^ T cells in blood and inflamed tissues of patients with autoimmune diseases, which are similar to Ly49^+^CD8^+^ T cells with regulatory function in mice. Consequently, CD8^+^ Tregs also exert their immunosuppressive function to inhibit autoreactive cells and maintain immune tolerance like what CD4^+^ Tregs do. However, the following questions need to be further investigated. What’s the mechanism on the generation and function of CD8^+^ Tregs? Do CD4^+^ and CD8^+^ Treg cells play a synergistic role in maintenance of immune tolerance?

### 2.4 B cells

Apart from the T cells, autoreactive B cells are also found in the joints of RA patients. Recent studies have identified an emerging subpopulation of B cells that are double-negative 2 (DN2) B cells induced by naive B cells and positively associated with disease activity. DN2 B cells-derived TNF-α accelerated the secretion of IFN-β and activation of RASFs via TNF-α mediated ERK1/2 and JAK-STAT1 pathways (66). DN2 B cells present its antigen to induce the polarization of Th17 and bone destruction (67). DN2 B cells are the major precursor to pathogenic antibody secreting cells (ASCs) in the RA synovium. ASCs secrete such auto-destructive factors as rheumatoid factor (RF) and ACPA, which are involved in the pathogenesis of RA (68). These antibodies can erode synovial tissue, synovitis and bone by forming immune complexes, stimulating the secretion of proinflammatory cytokines, and participating in the complement cascade or directly binding to osteoclasts (9, 69). As antigen-presenting cells, activated B cells facilitate T cell activation to secrete proinflammatory cytokines such as TNF-α, IL-6, IL-17 and IL-23, which induce inflammation and promote osteoclast differentiation. In RA patients, B cells also express chemokine CCL3 and TNF-α to inhibit osteoblast differentiation by activating the extracellular signal-regulated kinase (ERK) and NF-κB signaling pathways for joint injury (70). Conversely, it has been reported that B cells can also differentiate into regulatory B cells (Bregs) under the stimulation of proinflammatory cytokines. These Bregs secrete cytokines including IL-10 to suppress the function of Th1 and Th17 cells, reduce the levels of inflammatory cytokines secreted by DCs, and promote the differentiation of Forkhead Box P3 (Foxp3^+^) Tregs (71, 72). As subsets of Breg, B10 and B10 pro cells were converted to proinflammatory phenotype by TNF-α (73). Therefore, inducing Bregs is a potential treatment strategy for RA, and the synergistic effect of Bregs and Tregs is worth further exploration.

### 2.5 NK cells

NK cells, as member of the innate immune system, can interact directly or indirectly with other immune cells and participate in RA progress. The high CD38^+^ NK cells and low CD38^+^ NK-like T cells from the synovial fluid of RA patients suppress Treg cell differentiation by stimulating mTOR signaling in CD4^+^ T cells (74). Moreover, compared with non-deformable RA, NK cells from synovial fluids of patients with erosive deformable RA produced higher levels of TNF-α and IFN-γ upon IL-2 and IL-15 activation (75). As a result, the secreted TNF-α and IFN-γ further promote DC maturation to enhance T cell activation and differentiation. Additionally, NK cells facilitate DC antigen presentation by inducing apoptosis in target cell and releasing intracellular antigens, which promotes T cell activation. In RA patients, CD56^+^NKp44^+^ NK cells secret high level of IL-22 to promote the proliferation of fibroblast-like synoviocytes and lead to synovial hyperplasia, resulting in cartilage and bone destruction (76). Ogata et al. found that synovial NK cells co-cultured with monocytes can induce and promote the differentiation of monocyte lineages into osteoclasts, leading to bone and joint damage (5).

## 3 Treatment of RA

Traditional drug DMARDs including cyclophosphamide, sulfasalazine, methotrexate and NSAIDs are commonly used in the treatment of RA, although they can lead to some side effects and other functional impairments. TNF-α, IL-1 and IL-6 blockade and angiogenesis inhibition are also employed clinically due to their roles in the pathogenesis of RA (77). TCM has gradually emerged as a vital source of novel therapeutic agents for RA, attributed to its multifunctional bioactivities and ability to target multiple pathways. In addition, modern approaches like vaccines, nano-drug delivery systems, gene therapy and cell therapy have also been developed for RA treatment. The advantages and disadvantages of these therapies are summarized in **Table 1**.

**Table 1:** Therapies for RA treatment.

Therapies	Representative drug	Advantages	Disadvantages	Reference
**Traditional drug therapy**	Methotrexate	Inhibition of dihydrofolate reductase, the expression of Th1 cells and regulating RANKL-RANK-OPG pathway	Liver toxicity and low bioavailability	(78–80)
**Nano drug delivery system**	Gold nanoparticles	Binding to vascular endothelial growth factor and anti-angiogenesis	Poor pellet batch repeatability and difficult preparation scale	(81, 82)
**Biological agents**	Etanercept	Inhibition of TNF-α production, reduce the release of inflammatory cell mediators	Systemic toxicity, high cost	(83)
**Cell therapy**	CAR-T cell therapy	Targeting autoreactive cells and retention of protective cells	Relapse and immune rejection	(84)
**Traditional Chinese medicine**	*Tripterygium wilfordii Hook. f.*	Diverse active components, multiple targets, therapeutic effects on a variety of pathogenic cells	Heart, liver, digestive tract and bone marrow toxicity	(85)
**Vaccine**	Dendritic cell vaccine	Well tolerated and effectively regulating the balance of T cells and regulatory T cells	Expensive and time consuming	(86)
**Gene therapy**	Interferes with the IL-1βr gene	Reducing the expression of pro-inflammatory cytokines in arthritic joints and attenuating ankle swelling, bone erosion, and cartilage destruction	Expensive and time consuming, restriction of carrier vector	(87)

### 3.1 Traditional drug therapy

Methotrexate is the most commonly used DMARD due to its low cost and decades of clinical experience, and considered as the gold standard drug for RA treatment (88). Clinical practice guidelines often recommend the early initiation of methotrexate as an “anchor drug” in RA management (89–91). Methotrexate reduces tissue inflammation, slows down the destruction of tissue and cartilage, and has multipotent therapeutic effects on various immune cells and mediators (90, 91). The therapeutic mechanisms of methotrexate in RA include: inhibition of dihydrofolate reductase to reduce nucleic acid production and promote cell death (78), downregulation of Th1 cells and upregulation of Th2 cells and Tregs (79), amelioration of bone destruction by increasing osteoprotegerin (OPG) and decreasing RANKL (80), suppression of osteoclast differentiation (92), and inhibition of MMP expression and cartilage matrix absorption (93). However, with the increasing clinical application of methotrexate, side effects and deficiencies as intestinal inflammation and pulmonary fibrosis gradually appeared during the treatment. Moreover, methotrexate is easily eliminated by kidney to cause a shortened plasma half-life and low bioavailability. Furthermore, the concentration of methotrexate in target tissues can be reduced by macrophage phagocytosis (94). Finally, the pathophysiological and genetic differences among patients also limit its therapeutic efficacy for RA treatment (95).

Glucocorticoids have potent anti-inflammatory and immunosuppressive effects, and have been used to treat the acute phase of RA (96). A small dose of glucocorticoid can rapidly alleviate joint swelling and prevent joint bone destruction (97). However, long-term use of glucocorticoids is associated with decreased bone mineral density and increased fracture risk (96, 98), and raises the risk of infection and cardiovascular (99).

NSAIDs are one of the main therapeutic agents to alleviate the symptoms of RA. However, they do not modulate the underlying inflammatory immune responses or delay joint destruction (99). Long-term administration of high doses of NSAIDs often leads to adverse side effects, including indigestion, gastrointestinal bleeding or ulcers, high blood pressure, fluid retention and kidney damage. It is estimated that 5-7% of hospitalizations are related to adverse drug reactions. Approximate 30% of hospitalizations were due to NSAID-induced gastrointestinal, nervous, renal, or allergic reactions (100). Due to their potential toxicity, NSAIDs are usually introduced gradually or only when needed (99).

In addition to hormones, various small molecules such as cytokine inhibitors including TNF inhibitors (infliximab, etanercept, ardamuzumab) and IL-6 receptor antagonists (toximab), T cell activation modulators, and abbasiprol are now utilized clinically to treat RA (101). These biologic agents have made incredible breakthroughs in RA treatment and their therapeutic indices are fairly high in relation to non-biologic DMARDs. However, there are always two sides to everything, these modern research products inevitably have a variety of side effects depending on the choice and delivery methods of these agents, such as the risk of infection, malignancy, congestive heart failure, demyelinating disease, and hyperlipidemia (102, 103).

### 3.2 New treatment methods

With the advance of modern science, nano drug delivery systems have been developed to overcome the shortcomings of traditional drug therapies in terms of short retention time, low bioavailability and poor targeting. Along the same line, cell therapy, vaccine and gene therapy have also made great progress to improve therapeutic effects on RA treatment.

#### 3.2.1 Nano drug delivery system

Studies on nanomaterial drug delivery systems have shown that therapeutic agents coated with nanomaterials can change the overall drug particle size and maintain stable circulation in the blood to avoid their rapid removal from circulation by the reticuloendothelial system (RES) and excretion through glomerular filtration. Moreover, nano-carriers can selectively act on target cells without damaging other cells, reduce the dosage of drugs and improve their therapeutic effect (94). For example, when an injectable and pH-sensitive peptide hydrogel loaded with methotrexate and bismuthene nanosheet/polyethyleneimine (BiNS/PEI) reduced macrophage cytokine secretion and eliminated synovial fibroblasts, a multi-target therapeutic effect was achieved (104). Gold nanostars loaded with triamcinolones, when coupled with MSC to promote the repolarization of macrophages, can inhibit the Th17 differentiation and induce the regeneration of cartilage (105).

Nanotechnology or nanomaterials were also coated with cell membrane for RA treatment. The regulatory phenotype of FLS have potent inhibitory effects on T cells, but it is deficient in RA. Liu et al. designed the nanoparticles by coating cell membrane derived from regulatory FLS induced by IFN-γ plus rapamycin, demonstrating good efficacy, stability, and inflammatory joint targeting ability in RA (106). TNF-α and ROS exacerbate the synovial inflammation and tissue damage in joint of RA. Shan et al. constructed the nanocrystals including helical polypeptide, TNF-α siRNA and catalase, which were ultimately encased in macrophage cell membranes. When the nanomaterials accumulated in active joints, they degraded H_2_O_2_, inhibited the expression of TNF-α, and improved the targeting (107).

#### 3.2.2 Immune cell transfer

Since RA is an autoimmune disease in which immunological disorders play critical roles in its pathogenesis, adoptive transfer of genetically modified immune cells or their precursors can be good methods of treatment.

##### 3.2.2.1 CAR-T therapy

Chimeric antigen receptor T cells (CAR-T) are T cells that express multifunctional synthetic receptors through gene-editing techniques. Having achieved success in cancer therapy, this smart strategy also begins to demonstrate promising outcomes in the treatment of autoimmune diseases. Treg cell imbalance affects tolerance within the tissue, and RA patients have multiple pathogenic autoantigens, so CAR-Treg cells are further designed to improve tolerance to specific antigens. Citrullinated vimentin (CV) is an autoantigen exclusively in the extracellular matrix of the inflammatory synovial tissue in RA. It has been found that CV-CAR-Treg can promote the specific localization of Tregs and enhance their immunosuppressive function (108, 109). In addition, to replace current B-cell depletion therapies, the citrullinated antigen expressed by chimeric autoantibody receptor T cells (CAAR-T) has been used to delete anti-citrulline B cells while preserving protective B cells (84).

##### 3.2.2.2 Mesenchymal stem cell transplantation

MSC has multi-directional differentiation capacity into osteogenesis, fat etc, and secret various cytokines and miRNA-rich exosomes. MSC shows its immunosuppressive effect by directly or indirectly induces the polarization of CD4^+^ T cells to Tregs, and inhibits the proliferation of B cells and the production of antibodies. After intravenous injection of autologous bone marrow-derived MSCs, we found the expression of IL-4, IL-10, and TGF-β1 was increased by BM-MSCs through the inhibition of Th1 activity, thereby enhancing Treg function (110). In addition, MSCs inhibited the migration of DC to lymph nodes and suppressed the production of pro-inflammatory cytokines and chemokines. Furthermore, MSCs decreased the protein levels of TNF-α, CD83, CCR7, and MIP-1β in all monocyte subsets (111). Thus, MSC exerts multi-targeting and multi-functioning activities in RA treatment.

MCS can directly or indirectly regulates local matrix components through secreting vesicles or exosomes and promotes the repair of damaged joint tissues. It has been found that MSC derived vesicles rescued the frequency of Tregs, and suppressed the expression of IL-4, GM-CSF, IFN-γ, IL-2, TNF-α. Surprisingly, IFN-β-stimulated MSCs derived vesicles inhibited RA FLS migration and downregulated RA FLS surface markers (112). Moreover, MSCs derived exosomes induced Th2 and M2 polarization by enhancing TGF-β1 production. Similarly, MSCs-derived exosomes obtained from patient serum lessened cartilage damage by reducing TNF-α and IL-12 (113). In conclusion, treatment of MSCs with some agent can enhance the therapeutic effect. This review may help broaden our horizon for the therapy with MSC or its vesicles. These findings lay the foundation for the treatment of RA using MSCs or their vesicles.

#### 3.2.3 TCM treatment

##### 3.2.3.1 Treatment of synovial fibroblasts

TCM has a long history for the treatment of RA. Due to the complex composition, it’s hard to determine the major components, targets and mechanisms of TCM. With the progress of modern Chinese medicine, lots of active components have been isolated and characterized. Arecoline hydrobromide (AH) is a monomer alkaloid isolated from *Areca catechu L (* 114 *).* AH alleviated the pathological process of RA-FLSs by blocking the PI3K/AKT pathway (115). Coptisine inhibited the proliferation and migration of human fibroblast synovial cells by targeting PSAT1 and inhibiting the phosphorylation of p38, ERK1/2, and JNK (116). Osthole targeted AMPK, inhibited the secretion of inflammatory factors from FLS, restored mitochondrial homeostasis, and promoted the apoptosis in FLS by inhibiting the activation of NLRP3 (117).

##### 3.2.3.2 Treatment of bone damage

Joint bone erosion is in the latest stage of RA. Isopsoralen alleviated joint bone erosion and inhibited RANKL-induced osteoclast formation by blocking the NF-κB signaling pathway (118). Guizhi Shaoyao Zhimu decoction (GSZGs) reduced osteoclast precursors through the mitochondrial autophagic pathway (119). Moreover, isoimperatorin suppressed osteoclast formation by downregulating the expression of NFATc1 and inhibiting the combination of RANKL and RANK (120). Furthermore, boeravinone B (BOB) decreased the expression of osteoclast-specific gene NFATc1 by downregulating the RANKL/RANK signaling pathways, including NF-κB, MAPK, and PI3K/Akt. Notably, BOB also promoted the differentiation of bone marrow-derived MSCs into osteoblasts (121). An ethanolic extract from *Artemisia dracunculus* significantly increased osteogenic gene expression and increased mineralization (122). Similarly, as a positive regulator of osteogenesis, Bergamottin activated Wnt/β-catenin signaling pathway and increased the expression of osteoblast-specific gene (123).

In general, bioactive components of TCM can inhibit the proliferation and migration of fibroblast synovial cells, reduce osteoclast differentiation, bone resorption and cartilage degradation, and resolve the inflammation of RA. Therefore, they are potential candidates for RA treatment.

#### 3.2.4 Vaccine treatment

Vaccine can induce the expected immune responses to recover the inbalance of Th immune responses or immune overactivation caused by the autoimmune disease. Therefore, vaccination can be one of the most promising treatments for RA. A unique galactosylated collagen type II (COL2) peptide (Aq-galCOL2) binding to the MHC-II protein was used as a peptide-based vaccine. Complexes interacted directly with antigen-specific TCR via a positively charged tag, and led to the activation of unique unconventional regulatory T cells, resulting in a potent dominant suppressive effect (124). An inverse-vaccine, paKG(PFK15+bc2) microparticle balanced the populations of pro-inflammatory immune cells and Tregs, induced the generation of collagen specific anti-inflammatory T-cells, altered the oxidization of UDP-glucuronic acid and L-glutathione, and restored metabolic and immunological homeostasis in arthritic joints (125). Fibroblast activation protein (FAP) is expressed by RA synovial fibroblasts. A prophylactic mRNA-vaccine targeting FAP alleviated inflammatory response in arthritis mice (126). In addition, there are several other vaccines targeting auto-reactive T cells at clinical and experimental stages, such as b7-2/CD28 costimulatory signal vaccine (127) and human cartilage glycoprotein-39 vaccine, currently applicable in the clinics (128).

#### 3.2.5 Gene therapy

**Figure 5:**
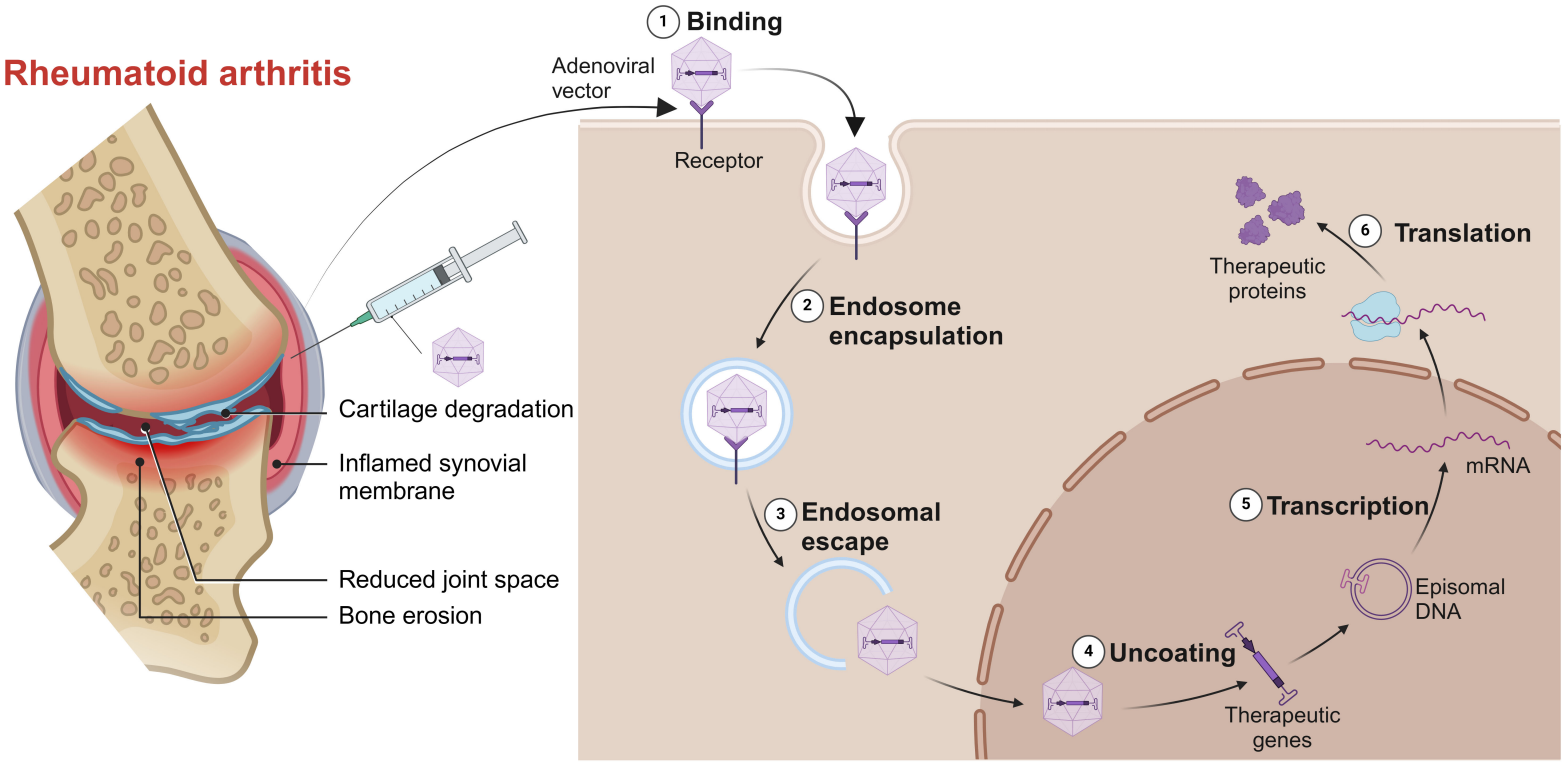
Gene therapy for RA. Intra-articular injection of vectors into the diseased joint, captured by the target cells, uncoated and transported into the nucleus to continuously express its products against pathogenic factors. Created with BioRender.com.

For those RA patients who can’t afford to wait for their immune responses against vaccines, gene therapy is a good option. Gene therapy involves intra-articular injection of vectors into the diseased joint to promote the apoptosis of target cells or continuously express their products against pathogenic factors (**Figure 5**). The engineered adeno associated virus (AAV) vectors loaded with anti-TNF-α gene had a long-term effects and alleviated inflammatory damage in the joint (129). Similarly, AAV/PD-L1 vectors decreased the levels of IL-6, IL-17 and TNF-α in joint, relieved joint edema and histopathological damage (130). The liposomes loaded with Pde3b siRNA polarized macrophage to M2 by enhancing PKA-CREB-C/EBPβ pathway, reduced inflammatory response, suppressed synoviocyte infiltration, and alleviated bone and cartilage erosion (131). Insulin like growth factor 2 mRNA-binding protein 2 (IGF2BP2) has antioxidant activity and plays a critical role in the regulation of autoimmune diseases. The AAV/IGF2BP2 attenuated paw edema, synovial hyperplasia and cartilage destruction by reducing the expression of MMPs *in vivo*, and inhibited the migration and invasion of RAFLSs (132). Other targets of gene therapy are summarized and shown in **Table 2**.

**Table 2:** Targets of gene therapy.

Relevant type	Vector type	Function	Reference
Calcium Release–Activated Calcium Channel 3	Adenovirus	Reduces the inflammatory and auto-immune responses, decreases the activity of mature osteoclasts	(133)
TNF alpha-induced protein 3	adeno-associated virus 6	Relieves the arthritis symptoms by inhibiting NLRP3, caspase-1, and IL-1β	(134)
Tumor necrosis factor-alpha-induced protein 8-like 2	Lentivirus	Attenuates collagen-induced joint severity by inhibiting the infiltration of macrophages and MDSCs	(135)
Caspase recruitment domain protein 6	Adeno-associated virus	Attenuates the severity of arthritis, improved histopathological damage, and hindered the bone erosion in collagen-induced arthritis (CIA) mice.	(136)
Aquaporin 1	Lentivirus	Affects the proliferation, migration and invasion of MH7A cells by Wnt/β-catenin signaling pathway,	(137)
β-arrestin-2	Adenovirus	Ameliorates ankle inflammation in CIA mice via NF-κB/NLRP3 inflammasome	(22)

With progressive understanding of RA pathogenesis, more and more targets for gene therapy will be identified with better specificity. Alone this line, a futuristic multigene transfer can be developed to aim multiple targets and pathways to achieve better therapeutic effect.

## 4 Conclusion

In general, the auto-reactive cellular immune responses including osteoclasts, synovial fibroblasts, T cells, B cells, NK cells, macrophages and DCs, play major roles in RA pathogenesis, although soluble inflammatory and rheumatoid factors with inflammatory natures were also involved. The imbalanced cellular responses and inflammation result in the proliferation of synovial fibroblasts, the injury of synovial cartilage, the differentiation and maturation of osteoclasts, and the inhibition of osteoblasts to cause bone degradation. Lots of drugs or strategies have been developed and applied on clinical trial (**Table 3**), such as DMARDs, biologic agents, TCM, cell transfer, gene therapy, vaccine and nano drug delivery system, to treat RA with both advantages and shortcomings. To have better therapeutic efficacy, the mechanism of RA pathogenesis, however, need to be further elucidated, especially the interaction among genetic background, environmental factors and immune system, so that effective drugs or therapeutic strategies can be developed to restore the tipped immune balance in RA.

**Table 3:** Different agents and their targets in clinical trial.

Classification	Agents	Targets	Phase	References
**Traditional drug therapy**	VC005 Tablets	JAK1	Phase 1	Clinicaltrials.gov
	LNK01001 Capsule	JAK1	Phase 2	Clinicaltrials.gov
**Nano drug delivery system**	TS-152	TNF-α and serum albumin	Phase3	Clinicaltrials.gov
	long-circulating liposomal prednisolone	synovial tissue	Phase 2	Clinicaltrials.gov
**Biological agents**	VDJ001	IL-6	Phase 2	Clinicaltrials.gov
	Abatacept	Memory B Cells	Phase4	Clinicaltrials.gov
**Cell therapy**	Autologous Tolerogenic Dendritic Cells	none	Phase 1	Clinicaltrials.gov
	Mesenchymal Stem Cell Secretome	none	Phase 1/Phase 2	Clinicaltrials.gov
**Traditional Chinese medicine**	2-HOBA	isolevuglandins	Phase 2	Clinicaltrials.gov
	Ursodeoxycholic Acid	synovial inflammation	Phase 2	Clinicaltrials.gov
**Vaccine**	COVID-19 Vaccine	none	Phase4	Clinicaltrials.gov
	Recombinant Herpes Zoster Vaccine	none	Phase4	Clinicaltrials.gov
**Gene therapy**	ART-I02	IFN-β	Phase 1	Clinicaltrials.gov
	tgAAC94	TNFR: Fc	Phase 1/Phase 2	Clinicaltrials.gov
